# Association between metabolic abnormalities and HBV related hepatocelluar carcinoma in Chinese: A cross-sectional study

**DOI:** 10.1186/1475-2891-10-49

**Published:** 2011-05-15

**Authors:** Jinyan Zhao, Yunpeng Zhao, Hao Wang, Xing Gu, Jun Ji, Chunfang Gao

**Affiliations:** 1 Department of Laboratory Medicine, Eastern Hepatobiliary Surgery Hospital, Second Military Medical University, Shanghai, China; 2 Department of Laboratory Medicine, Changzheng Hospital, Second Military Medical University, Shanghai, China

## Abstract

**Background:**

Previous studies suggested that the abnormality of metabolism is a newly identified risk factor in HBV-related hepatocellular carcinoma (HCC). The association between metabolic factors and hepatocellular carcinoma (HCC) has not been clarified up to now. This study was conducted to investigate the prevalence of metabolic abnormalities in HCC and to probe the association between metabolic parameters and liver function as well, so as to evaluate the interactions between metabolism and the development of HBV-related HCC.

**Methods:**

Totally 179 cases of HBV-related HCC, who were surgically treated and pathologically confirmed were enrolled. HBV carriers (n = 100) and healthy controls (n = 150) were recruited from routine physical examination during the same period. Body mass index (BMI) was obtained from medical documentation. All the metabolic-related parameters and liver function tests were determined with routine biochemical or immunological analytic methods. Malondialdehyde (MDA) and total antioxidant capacity(TAOC)were detected by chemical analytic methods. A stratified analysis was conducted according to BMI, glycated albumin (GA), free fatty acids (FFA), and the relationships between the metabolic-related parameters and liver functions were analyzed in HCC and control subjects.

**Results:**

HCC group showed significantly high levels of mean BMI, serum glucose, low serum lipids levels than controls (P < 0.05). Acquired by stratified analysis, the higher the BMI, the higher level of insulin and homeostasis model assessment for insulin resistance (HOMA-IR) (P < 0.01) were found in HCC patients. Elevated level of MDA and γ-glutamyltransferase (GGT) were revealed in those with high serum FFA level for the first time. Strong associations between metabolic factors and liver function were shown in HCC (P < 0.05). Higher GA level was strongly associated with increased risk of cancer compared to healthy controls (OR = 9.87, 95% confidence interval: 1.86~52.29). Serum triglycerides (TG) and low-density lipoprotein cholesterol (LDL-C) levels were negative contributory factors for HCC (OR = 0.05, 95% confidence interval: 0.01~0.27 and OR = 0.32, 95% confidence interval, 0.11~0.95: respectively).

**Conclusions:**

Metabolic abnormalities are closely associated with the occurrence and development of HBV-related HCC. Oxidative stress and/or lipid peroxidation might be involved in the pathogenesis and acceleration of liver function impairments in HCC.

## Background

Hepatocellular carcinoma (HCC) is the fifth most common cancer and the third cause of cancer-related death worldwide [1]. It has been a major concern in both Western and Asia countries. As known to all, the high prevalence of hepatitis B and C gives rise to the high incidence of HCC. At the same time, so many confounding factors are associated with the occurrence and development of chronic liver diseases [2]. Recently, the relationship between metabolic factors and chronic liver diseases including liver cirrhosis (LC) and hepatocellular carcinoma (HCC) has become a hot topic [3].

Metabolic syndrome (MS) has been recognized as a major public health problem worldwide arousing more attentions. MS is a collection of metabolic abnormalities, including abdominal obesity, blood lipid barrier, diabetes, hypertension. MS is interrelated with insulin resistance, which is also known as insulin resistance syndrome [4]. Nonalcoholic fatty liver disease (NAFLD) as the hepatic manifestation of MS, has been revealed to be associated with insulin resistance [5]. NAFLD is no longer a disease happened in developed Western countries. Fan, et al reported that the prevalence of NAFLD is up to 15% in some urban of China [6]. It was described as a young disease and could progress to end-stage liver diseases, from simple fatty liver, steatohepatitis to liver cirrhosis and HCC [7,8].

Laboratory tests are useful in reflecting the metabolic abnormalities or liver function impairments. Abnormal levels of aminotransferase (ALT) and bilirubin usually indicate liver functions impairment, but the metabolism of lipid or blood glucose is also among the important functions of the liver. Recently some researches reported that γ-glutamyltransferase (GGT) and ALT could predict the development of MS [9,10]. Although the associations between metabolic factors and hepatocellular carcinoma (HCC) have been gradually recognized, fewer investigations have been made between the metabolic indicators and HBV-related HCC. Making use of the high prevalence of HBV-related HCC in China [11], we designed a cross-sectional study to clarify the association between metabolic abnormalities and the development of HBV-related HCC.

## Methods

### Subjects and measurements

The study consisted of 179 cases of patients with HBV-related HCC who were diagnosed and confirmed by pathology in the Shanghai Eastern Hepatobiliary Surgery hospital (EHBH) from January to August 2008. Liver cirrhosis was revealed in 66.5% (119/179) of HCC patients. Serum HBsAg was positive in all enrolled HCC. The HBeAg positive cases accounted for 66.5% (119/179). The level of serum HBV DNA higher than 10^3 ^copy/ml accounted for 63.7% (114/179). The HCC stage was classified according to the TNM criteria (2002) [12]: T1, a solitary tumor without vascular invasion; T2, a solitary tumor with vascular invasion or multiple tumors of 5 cm or less; T3, multiple tumors greater than 5 cm invading the major branches of portal or hepatic veins; and T4, tumors with direct invasion of adjacent organs other than the gallbladder or with perforation of visceral peritoneum. N0/1, with/without regional lymph node metastasis. M0/1, with/without distant metastasis. Stage I, T1N0M0; Stage II, T2N0M0; Stage IIIA, T3N0M0; Stage IIIB, T4N0M0; Stage IIIC, Tany N1M0; Stage IV, Tany Nany M1. All the patients' information was obtained by hospital medical records. One hundred cases of HBV carriers were collected from the Shanghai Changzheng hospital. The HBV carriers was defined as positive serum hepatitis B surface antigen (HBsAg) for at least 6 months but who have repeatedly normal (or minimally raised) liver enzymes and negative tests for HBeAg before enrolling [13]. Patients with hepatitis A virus, HCV, hepatitis D virus, hepatitis E virus, human immunodeficiency virus, Epstein-Barr virus, and cytomegalovirus infection, alcohol consumption > 30 g/day, metastatic liver cancer, autoimmune liver disease, drug-related liver disease, alcoholic hepatitis, obstructive jaundice, other causes of chronic liver disease, renal inadequacy or blood diseases were excluded from the patients cohort. The 150 cases of healthy control were recruited from routine physical examination in EHBH.

The study protocol was approved by the Chinese Ethics Committee of Human Resources, Eastern Hepatobiliary Hospital. Informed consents were obtained both from the patients and the healthy donors.

The information of weight, height, blood pressures, tumor size and whether there were any violations of metastasis, medical history, life style characteristics and other related information were collected from medical documentation. Body mass index (BMI) was calculated by dividing body weight in kilograms by the square of height in meters. Waist circumference was not measured in this study. According to the standard of obesity in Chinese population [14], obesity was divided into two groups, normal BMI level (BMI 18.5 to 23.9 kg/m^2^) and abnormal BMI (BMI≥ 24 kg/m^2^). Blood specimens were drawn after 12 h of fasting and were subsequently measured at the Department of Laboratory Medicine in EHBH. All the blood samples were collected before surgery. The measured liver function indicators were: total bilirubin (TBIL), direct bilirubin (DBIL), total bile acid (TBA), total protein (TP), albumin (ALB), prealbumin (PALB), ALT, aspartate aminotransferase (AST), GGT, alkaline phosphatase (ALP). Indicators of lipid metabolism consisted of FFA, TG, total cholesterol (TC), LDL-cholesterol (LDL-C), apolipoprotein B (ApoB). Relevant indicators of glucose metabolism included glycated albumin (GA), fasting glucose, insulin and C-peptide. All the measurements were performed on HITACHI 7600 automatic biochemical analyzer with matched reagents. High-density lipoprotein cholesterol (HDL-cholesterol, HDL-C) was calculated by the Friedewald equation [15]. Quantitative insulin sensitivity check index (QUICKI) [16] and homeostasis model assessment for insulin resistance (HOMA-IR) were calculated to evaluated insulin resistance [17]. Oxidative stress marker MDA was measured using the thiobarbituric acid-reactive substances (TBARS) assay, TAOC was measured with Fe^3+ ^reduction method. Alpha-fetoprotein (AFP), carcino-embryonic antigen (CEA) and CA19-9 were determined on Roche E170 with chemiluminescence; Hepatitis B virus related surface antigen and antibody were detected by ARCHITECT I 2000SR using chemiluminescent microparticle immunoassay (CMIA).

### Statistical analysis

The statistical analyses were conducted using SPSS version 11.5 statistical software Data were presented as median and range for nonparametric variables and mean ± SD for parametric variables. One-way analysis of variance for non-parametric data was performed by a post hoc analysis using Kruskal-Wallis H test. Parametric variables were compared by post hoc analysis using Tukey test. BMI, GA, FFA were classified into categories so as to make stratified analysis. Categorical variables were compared using a Mann-Whitney U test. For analysis of the correlation between two indicators (liver functions and metabolic parameters), spearman correlation analysis was performed. For multivariate analysis, multivariate logistic regression analysis was applied. The OR was used to estimate the relative risk of HCC. All p-values were two-tailed and p-values less than 0.05 were considered significant.

## Results

**Figure 1:**
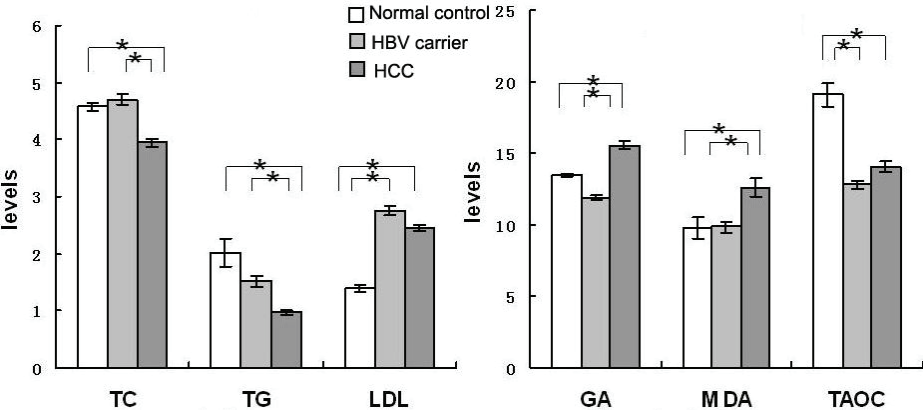
**Metabolic parameters in healthy controls, HBV carriers and HCC**. Totally 179 cases of patients with HBV-related HCC, 100 cases of HBV carriers and 150 cases of healthy controls were recruited. The liver function indicators and GA were measured on HITACHI 7600 automatic biochemical analyzer with matched reagents. Oxidative stress marker malondialdehyde (MDA) was measured using the thiobarbituric acid-reactive substances (TBARS) assay, total antioxidant capacity (TAOC) was measured with Fe^3+ ^reduction method. HCC group showed significantly lower levels of TC, TG and higher levels of GA than both diseased and healthy controls (p < 0.01), while significantly higher levels of MDA and lower levels of TAOC were revealed in HCC (p < 0.01). LDL-C was higher in HCC than that in healthy controls (p < 0.01). Data were expressed as percentages ± standard error. * *p *value < 0.01 vs. control. Parametric variables were compared by post hoc analysis using Tukey test.

The general demographic information of all the subjects was listed in Table 1. There were significant differences in TBIL, TP, ALB, ALT, AFP among HCC patients, HBV carrier and healthy controls (p < 0.05). Stages of HCC were 49 (27.37%) in I, 69 (38.55%) in II, 49 (27.37%) in III and 6 (3.35%) in IV by TNM classification. The metabolic parameters of the three groups were displayed in Figure 1. HCC group showed significantly lower levels of TC, TG and higher levels of GA than both diseased and healthy controls (p < 0.01), while significantly higher levels of MDA and lower levels of TAOC were revealed in HCC (p < 0.01). Though there was no difference for LDL-C between HCC ptients and HBV carriers, LDL-C was higher in HCC patients than that in healthy controls (p < 0.01).

**Table 1:** Characteristics of all enrolled subjects

Indices	Healthy (X ± SD)	HBV(X ± SD)	HCC(X ± SD)	F	p
AGE	47.48 ± 8.05	46.85 ± 12.06	52.45 ± 9.85	15.89	0.00
Sex	Male(118)	Male(66)	Male(155)	16.43	0.00
	Female(32)	Female(34)	Female(24)		
TBIL(μmol/L)	12.64 ± 5.08	15.54 ± 8.67	16.38 ± 14.16	4.709	0.009
TP(g/L)	77.71 ± 3.25	78.49 ± 4.90	71.23 ± 6.04	98.155	0.00
ALB(g/L)	47.20 ± 2.24	48.27 ± 2.21	40.91 ± 3.96	250.025	0.00
ALT (U/L)	22.46 ± 15.00	31.33 ± 20.37	54.13 ± 65.15	20.005	0.00
AFP(μg/L)	2.8(1.82-3.88)	1.54(1.13-2.28)	34.4(5-1210)	149.83	0.00

Data were presented as mean ± SD, or median and range. Differences in continuous variables were evaluated by One-way analysis of variance with post hoc analysis using Tukey test and Kruskal-Wallis test.

* *p *value < 0.05 was considered statistically significant.

The impacts of BMI, GA, FFA on liver functions as well as on the other metabolic parameters were revealed in Table 2. With the increasing of BMI, the higher level of GLU, HOMA-IR, Insulin, QUICKI, FFA, TG and LDL-C. (p < 0.05) were found in HCC patients. There was no significant difference of liver function in different BMI subgroups. GLU and ALP were significant higher in higher GA group than that in normal GA after stratified by GA at the borderline value (p < 0.05). Similarly, FFA was used to classify the subjects into abnormal (higher) and normal group, MDA and GGT were significantly higher in abnormal group than that in normal one (p < 0.05). We failed to find any differences between the TNM stages and the metabolic parameters or liver functions (data not shown).

**Table 2:** Changes of metabolic parameters and liver functions in HCC subjects with different BMI, GA, FFA

	BMI(median)		P	GA(median)		P	FFA(median)or (X±SD)		P
	**<24 kg/cm^2^**	**≥24 kg/cm^2^**		**≤16%**	**>16%**		**≤0.6**	**>0.6**	

BMI (kg/m2)				22.6 (20.70~25.19)	23.24 (21.15~24.67)	0.86	22.45 (21.68~24.73)	24.67 (20.62~26.08)	0.45
GA	15(14~16)	16(13~16)	0.08				15.5(14~16)	14.5(14~16)	0.18
GLU (mmol/L)	4.96(4.48~5,23)	5.2(4.68~5.39)	0.03	4.86 (4.54~5.1)	6.43(5.06~6.59)	0.00	5.03(4.59~5.38)	5.17(4.67~5.39)	0.64
INS (μU/L)	7.46(3.79~7.47)	7.77(5.15~10.18)	0.00	7.46(3.97~8.93)	9.06(3.92~10.03)	0.81	7.32 (4.15~9.16)	8.32(4.29~10.38)	0.29
C-peptide (nmol/L)	1.18(0.84~1.39)	1.18(1.13~1.95)	0.00	1.16(0.91~1.55)	1.26(0.96~1.53)	0.92	1.18(1.0~1.56)	1.24 (1.16~2.0)	0.04
IR	1.53(0.8~1.62)	1.9(1.11.~2.54)	0.00	1.43(0.87~2.03)	3.16(0.90~2.44)	0.07	1.5(0.90~2.06)	2.59 (1.12~2.41)	0.11
QUICKI	0.36(0.35~0.39)	0.35(0.33~0.37)	0.00	0.36(0.34~0.39)	0.32(0.33~0.39)	0.07	0.36(0.34~0.38)	0.33(0.33~0.37)	0.11
FFA (mmol/L)	0.39(0~0.54)	0.61(0~0.58)	0.03	0.46(0~0.56)	0.5(0.1~0.57)	0.32			
TC(mmol/L)	3.60(3.31~4.42)	4.44(3.55~4.71)	0.04	3.84(0.35~0.46)	4.26(3.42~4.49)	0.59	3.83(3.4~4.48)	4.32(3.63~4.67)	0.36
TG(mmol/L)	0.79(0.61~1.02)	1.20(0.83~1.31)	0.00	1.17(0.68~1.17)	0.79(0.64~1.05)	0.38	1.05(0.73~1.17)	1.12(0.8~1.21)	0.63
LDL(mmol/L)	2.24(1.92~2.64)	2.77(2.2~3.04)	0.00	2.42(1.93~2.86)	2.61(1.95~2.77)	0.68	2.39(1.96~2.87)	2.4(2.02~2.89)	0.70
HDL(mmol/L)	1.31(1.14~1.59)	1.38(1.09~1.48)	0.04	1.32(1.08~1.48)	1.35(1.22~1.63)	0.03	1.33(1.07~1.49)	1.35(1.19~1.53)	0.46
MDA(nmol/mL)	10.75(7.48~15.8)	10.88(6.22~14.05)	0.34	10.63(6.97~15.38)	12(6.38~14.73)	0.98	11.24 ± 6.74	15.03 ± 12.33	0.03
TAOC(U/ml)	12.03(10.64~17.13)	15.36(10.36~18.19)	0.21	12.46(10.47~17.04)	16.84(11.63~18.53)	0.05	11.96(9.22~17.51)	14.4(10.85~17.82)	0.20
ALB(g/L)	41.6(38.45~43.63)	39.6(38.6~43.2)	0.84	41.6(39.04~43.7)	39.2(37.2~42)	0.02	41.8(38.74~43.49)	39.95(37.13~42.08)	0.10
ALT(U/L)	30.2(26.53~55.93)	44.5(26.5~53.4)	0.89	30.2(25.95~53.75)	49.9(32.15~56.7)	0.27	38.85(25.65~56.65)	31.95(29.18~56.35)	0.72
AST(U/L)	34.6(29.68~55.68)	34.9(25.4~58.2)	0.10	33.5(26.43~53.53)	44.6(29.65~64.25)	0.12	34.85(27.2~51.5)	31.62(30.03~66.75)	0.14
ALP(U/L)	97(76~122)	79(63~105)	0.06	84(67~110.25)	112(82~144)	0.01	88.5(70.5~113)	89(74.5~159.25)	0.07
GGT(U/L)	77(40~171)	55(36~123)	0.30	65(36~135)	77(42~109)	0.60	62(35.5~99)	144(81~215.5)	0.00

Data were presented as median and range. Differences were regarded as significant at 0.05 in a Mann-Whitney U test.

*p value < 0.05 was considered statistically significant.

The association study revealed that the parameters reflecting liver function were correlated with metabolic indicators (Table 3). Especially the liver enzymes, AST and ALP, were positively correlated with QUICKI, GA, INS, (p < 0.05) and negatively correlated with IR. GGT was correlated with FFA and HDL-C (p < 0.05). The association study among metabolic parameters in HCC patients showed that BMI was positively associated with IR, INS, C-peptide and negatively related with QUICKI and GA. MDA was positively associated with FFA and negatively related with HDL. TAOC was correlated with TC, TG and HDL-C (p < 0.05) (Table 4).

**Table 3:** Association study among liver functions, tumor markers and metabolic parameters in HCC

		BMI	IR	QU	GA	GLU	INS	C-peptide	FFA	TC	TG	LDL	HDL
TBIL	r	-0.034	0.02	-0.02	0.092	0.018	0.019	0.043	0.188	0.091	-0.228	0.032	0.236
	p	0.663	0.8	0.8	0.222	0.816	0.806	0.573	0.036	0.23	0.002	0.0675	0.002
ALB	r	0.018	0.186	-0.186	-0.248	-0.019	0.196	0.08	-0.08	0.165	-0.016	0.189	-0.009
	p	0.813	0.015	0.015	0.001	0.8	0.01	0.296	0.377	0.029	0.838	0.012	0.902
ALT	r	-0.03	-0.013	0.013	0.08	0.102	-0.057	0.056	0.103	-0.024	0.064	-0.057	0.018
	p	0.694	0.863	0.863	0.289	0.175	0.459	0.467	0.252	0.753	0.403	0.449	0.814
AST	r	-0.183	-0.217	0.217	0.188	-0.02	-0.241	-0.079	0.161	0.051	-0.073	-0.007	0.141
	p	0.017	0.004	0.004	0.013	0.79	0.002	0.305	0.073	0.502	0.339	0.931	0.062
ALP	r	-0.204	-0.25	0.25	0.272	0.043	-0.302	-0.156	0.125	-0.054	-0.012	-0.125	0.111
	p	0.008	0.001	0.001	0	0.571	0	0.042	0.166	0.476	0.879	0.098	0.143
GGT	r	-0.099	-0.102	0.102	0.063	-0.036	-0.136	0.033	0.311	0.1	0.063	0.035	0.174
	p	0.248	0.232	0.232	0.452	0.665	0.11	0.699	0.002	0.235	0.459	0.675	0.037
PT	r	-0.063	-0.175	0.175	0.231	-0.089	-0.158	-0.15	0.107	-0.29	-0.242	-0.317	-0.084
	p	0.412	0.022	0.022	0.002	0.237	0.037	0.049	0.236	0	0.001	0	0.266
AFP	r	-0.071	-0.091	0.091	-0.054	-0.131	-0.065	-0.108	0.069	-0.007	-0.115	-0.005	0.002
	p	0.35	0.232	0.232	0.475	0.08	0.393	0.159	0.443	0.924	0.13	0.949	0.974

The correlation between two indicators (liver functions and metabolic parameters), spearman correlation analysis was performed.

* *p *value < 0.05 was considered statistically significant.

**Table 4:** Association study between metabolic parameters in HCC

		BMI	FFA	TC	TG	LDL	HDL
IR	r	0.391	0.208	0.122	0.179	0.14	-0.095
	p	0.000	0.023	0.109	0.019	0.067	0.214
QUICKI	r	-0.391	-0.208	0.122	-0.179	-0.14	0.095
	p	0.000	0.023	0.109	0.019	0.067	0.214
GA	r	-0.21	0.133	0.005	-0.218	0.121	0.311
	p	0.006	0.138	0.947	0.004	0.107	0.000
GLU	r	0.117	0.081	0.032	0.127	0.044	-0.033
	p	0.127	0.368	0.67	0.094	0.563	0.665
INS	r	0.416	0.146	0.133	0.148	0.155	-0.082
	p	0.000	0.112	0.08	0.054	0.042	0.282
C peptide	r	0.376	0.194	0.128	0.205	0.152	-0.104
	p	0.000	0.034	0.094	0.007	0.046	0.171
MDA	r	-0.062	0.213	-0.13	-0.035	0.081	-0.168
	p	0.421	0.019	0.086	0.648	0.287	0.026
TAOC	r	0.113	0.083	0.172	0.224	0.112	0.155
	p	0.151	0.377	0.025	0.003	0.146	0.043

The correlation between two indicators (metabolic parameters), spearman correlation analysis was performed.

* *p *value < 0.05 was considered statistically significant.

GA, TG, LDL and HDL were shown as risk factors for HCC after stepwise multiple regression analysis (Table 5). High GA level was strongly associated with increased risk of cancer compared to healthy control (OR, 9.87; 95% CI, 1.86~52.29) and HBV carriers (OR, 16.67; 95% CI, 1.89~147.3). Interestingly serum TG levels were strongly associated with reduced risk of cancer development (OR = 0.05, 95% CI: 0.01~0.27) and HBV carriers (OR = 0.12, 95% CI: 0.04~0.37) respectively. The risk of cancer was also markedly attenuated with higher LDL-C levels (OR = 0.32, 95% CI: 0.11~0.95). Low HDL-C was strongly associated with increased risk of HBV carriers (OR = 11.81, 95% CI: 2.37~58.91) and HCC development (OR = 11.99, 95% CI: 1.8~77.56) respectively.

**Table 5:** Multiple logistic regression analysis to explore risk factors contributing to HCC

	HCC vs controls OR(95%CI)	P values	HCC vs HBV carrier OR(95%CI)	P values	HBV carrier vs controls OR(95%CI)	P values
GA						
≧16	9.87 (1.86~52.29)	0.007	16.67(1.89~147.3)	0.011	0.60 (0.05~7.15)	0.680
TG(mmol/L)						
≧2.29	0.05 (0.01~0.27)	0.001	0.40 (0.07~2.20)	0.293	0.12 (0.04~0.37)	0.000
LDL(mmol/L)						
≧3.11	0.32 (0.11~0.95)	0.040	0.80(0.25~2.57)	0.705	0.4 (0.17~0.96)	0.040
HDL(mmol/L)						
<0.92	11.99 (1.8~77.56)	0.090	1.02 (0.25~4.10)	0.983	11.81 (2.37~58.91)	0.003

Multivariate logistic regression analysis was applied.

* *p *value < 0.05 was considered statistically significant.

The above results were also achieved after stratified analysis by age (see Additional file 1 Table S1-S3) and gender (see Additional file 2 Table S4-S5). The tumor size was negatively associated with TG (p < 0.05) and positively related to GGT (p < 0.01) (see Additional file 3 Figure S1).

## Discussion

Metabolic syndrome is composed of a complex constellation of factors, that namely glucose intolerance and insulin resistance, central obesity, dyslipidemia, hypertension [4,18,19]. Over the past decade several researches have produced preliminary clues on the relationship between metabolic syndrome and hepatocellular carcinoma [20,21]. However, few studies have investigated the association between metabolic factors and HBV-related HCC. As with the development of economy and changing of living behavior, metabolic-related chronic diseases have become more common in China. The present study extended the research on metabolic abnormalities in HBV related HCC in Chinese populations.

Insulin resistance and obesity, the majority components of MS are often revealed to be linked with cancer and now widely recognized as significant risk factors for HCC [22-24]. Our data displayed that with the increasing of BMI, the higher level of GLU, HOMA-IR, Insulin, QUICKI, FFA, TG and LDL-C. (p < 0.05) were found in HCC. Similar study demonstrated that HOMA-IR, a marker of insulin resistance, is elevated with increasing BMI[25]. The link between obesity/diabetes and HCC may be explained by oxidative stress and/or lipid peroxidation that increase the possibility of hepatic injury, fibrosis, and even cirrhosis result from hepatic inflammation [26,27]. Increased plasma insulin concentration combined with impaired whole-body glucose clearance in the present study was most likely a consequence of decreased insulin sensitivity, a typical phenomenon in patients with malignant diseases [28,29].

In investigation the existence of oxidative stress and/or lipid peroxidation in HCC, we measured MDA and TAOC in all subjects. MDA is the lipid peroxidation end product and an important player in oxidative stress. Now MDA is widely used as one of the most reliable indices of oxidative stress to evaluate the oxidative damage in diseased status[30]. TAOC is an index of the antioxidative defense system. We found for the first time that elevated levels of oxidative stress(MDA) and decreased antioxidative capacity (TAOC) were revealed in HCC patients. The results were consistent with the finding that HBV infection could increase toxic oxidation products, which lead to oxidative stress and DNA damage [31,32]. HBV X protein has drawn considerable attention to the generation of HCC via mitochondria-derived reactive oxygen species (ROS). ROS could activate kupffer cells modulating hepatocyte injury and generating ROS, accompanied by lipid peroxidation, further impairment of mitochondrial function, which contributed much to carcinogenesis [32]. In addition, ROS are most potent and can modify DNA methylation [33,34]. But what predominates the extent of liver injury in HBV-related HCC and how abnormal metabolism accelerates the pathogenesis of HCC remain to be elucidated.

The liver is a critical organ in maintaining glucose and lipid homeostasis. The decreased glucose metabolism, utilization and downregulation of triglyceride synthesis in HCC patients were revealed in this study. ALT as an indicator of liver injury was obviously elevated in HCC in our observation (table 1). The elevation of ALT levels might be the reflection of excess accumulation of fat in the liver because of various metabolic abnormalities and indicated ongoing inflammation which impairs insulin signaling in the liver [35-37]. Our further logistic regression analysis revealed that lower TG and cholesterol levels as well as higher GA were contributing factors to HCC (Table 5). Meanwhile we found that the inverse correlation between serum TG and tumor size, the positive correlation between tumor size and GGT (supplementary Figure 1). These interesting observations indicate the intimate relation between lipid or glucose metabolisms and HBV-related malignancies, The contradicatory associations, ie. the inverse and positive correlations between lipid metabolisms and HCC, as revealed by us and others further demonstrated the complicated process of dyslipidemia involved in the pathogenesis HCC[26,38,39]. Though the mechanism how HBV-related diseases were closely related to lipid metabolisms is not clarified up to now, there are several studies supporting our findings: (1) HBV infection disturbs liver lipid metabolism, which was reported by Jan et al and revealed that low serum TG level was also associated with chronic HBV infection [40]. Another independent in vitro study had ever demonstrated that HBX protein could directly inhibit secretion of ApoB, an essential component of LDL-C and very-low-density lipoprotein (VLDL-C), via promoting high expression of N-acetylglucosaminyltransferase III, in cell culture [41,42]. (2) Lipids and lipoprotein metabolisms could be regulated by cytokines, while tumor cells are known to produce large amounts of pro-inflammatory cytokines. For instance, interleukin-6 (IL-6), tumor necrosis factor (TNF-α), IL-1 may inhibit TG synthesis [43]. (3)The change of general nutritional status in exhaustive malignant diseases is another important factor leading to dyslipidemia. Low levels of TG and cholesterol were also revealed in other malignancies including colorectal cancer, lung cancer and so on [44-47]. Although our findings of inverse relationship between TG level and HCC were also consistent with previous study[26,48], the possibility that low TG was a consequence of malignant liver diseases (HCC) can not be excluded. Hence further prospective study should be designed to elucidate the causative or consequent effect or even both causative and consequent effects of dyslipidemia in HBV-related HCC in future study.

Another important metabolic indicator which could be linked with obesity is FFA. FFA is normally released from an expanded adipose tissue and produced an increased secretion of VLDL. MDA, the lipid peroxidation end product via fatty acid β-oxidation, was found to be associated with FFA in this study. Our result further confirmed that obesity might be related with liver injury and eventual accelerating the development of HCC [49,50].

There are some limitations of this study. HBV carrier was used as disease control instead of CLD (fibrosis and cirrhosis). Metabolic abnormality might develop in CLD and abnormal metabolism could accelerate the progress of CLD at the same time. Further validation in CLD groups and in large sample size were required to illustrate the value of metabolic assessment in the prediction and prognosis in HCC. Another limitation is that the research is a cross-sectional study drawn from a clinical series of patients and not from the community. But the cases were carefully matched or stratified by age, gender, BMI, time of hospital admission and etiology to minimize the interferences. A better understanding of the relationship between these metabolic parameters should ultimately lead to improve prediction and treatment options for patients with HBV related HCC. To counteract the adverse effects of metabolic abnormalities, those infected with HBV should first attempt to achieve the metabolic control through changes in diet and life style.

## Conclusions

This study concluded that metabolic abnormalities are closely associated with the occurrence and development of HBV-related HCC. Oxidative stress and/or lipid peroxidation identified in HCC might be involved in the pathogenesis. The predictive value of metabolism parameter and the pathogenesis of abnormal metabolism in HCC require further validation in future study.

## List of abbreviations

(BMI): Body mass index; (MDA): malondialdehyde; (TAOC): total antioxidant capacity; (GA): glycated albumin; (Ins): Insulin; (QUICKI): Quantitative insulin sensitivity check index; (HOMA-IR): homeostasis model assessment for insulin resistance; (FFA): free fatty acids; (ROS): reactive oxygen species; (TBIL): total bilirubin; (DBIL): direct bilirubin; (TBA): total bile acid; (TP): (ALB): total protein; albumin; (ALT): alanine aminotransferase; (AST): aspartate aminotransferase; (GGT): γ-glutamyltransferase; (ALP): alkaline phosphatase; (TG): triglycerides; (TC): total cholesterol; (LDL-cholesterol): low-density lipoprotein cholesterol; (HDL-cholesterol): High-density lipoprotein cholesterol; (ApoB): apolipoprotein B; (AFP): alpha-fetoprotein.

## Competing interests

The authors declare that they have no competing interests.

## Authors' contributions

JYZ carried out the patients enrollment, biochemistry analysis and drafted the manuscript. YPZ carried out the biochemistry and antioxidant determinations. HW participated the subjects enrollment and part of the biochemistry detections. XG participated the MDA determination and statistic analysis. JJ participated part of the biochemistry analysis. CFG was responsible for the study design, data analysis and manuscript modification. All authors read and approved the final manuscript.

## Supplementary Material

Additional file 1 **Stratum analysis of parameters in accordance with age**. After stratified by age, the results were similar to what we have got from the whole subjects. Click here for file

Additional file 2 **Stratum analysis of parameters in accordance with gender**. After stratified by gender, the results were similar to what we have got from the whole subjects. Click here for file

Additional file 3 **The associaton between tumor size and TG or GGT**. Correlation analysis between tumor size and TG. The tumor size was negatively associated with TG and positively related to GGT. Click here for file
