# Supplementary material for: Association between metabolic abnormalities and HBV related hepatocelluar carcinoma in Chinese: A cross-sectional study
Source: Nutr J. 2011 May 15;10:49. doi: 10.1186/1475-2891-10-49 (PMC3118330; doi:10.1186/1475-2891-10-49)
Supplement: Additional file 1 — Stratum analysis of parameters in accordance with age. After stratified by age, the results were similar to what we have got from the whole subjects. [file 1475-2891-10-49-S1.PDF]

Additional file 1(Table S1- Table S3)

Table S1

Stratum analysis of different parameters in all subjects younger than 50-year-old

Indices	healthy(91)	HBV(58)	HCC(69)	F	P
GA	13.42±1.34	11.64±1.36	14.52±2.28	45.95	0.000
TC (mmol/L)	4.44±0.73	54.70±0.99	3.87±0.88	16.00	0.000
TG (mmol/L)	2.00±1.48	1.62±0.89	0.90±0.34	15.29	0.000
LDL (mmol/L)	2.87±0.69	2.80±0.80	2.44±0.74	7.40	0.001
HDL (mmol/L)	1.28±0.26	1.41±0.41	1.25±0.34	3.68	0.027
MDA (nmol/mL	9.08±5.59	9.97±4.97	13.17±6.52	10.62	0.000
TAOC (U/ml)	19.15±17.06	5.46±2.71	14.04±4.97	25.09	0.000
TBIL(μmol/L)	12.74±4.83	15.74±9.22	14.07±5.59	3.74	0.025
TP(g/L)	78.04±3.27	78.67±4.88	70.95±5.52	59.64	0.000
ALB(g/L)	47.41±2.26	48.35±1.75	41.02±3.51	162.91	0.000
ALT (U/L)	25.90±17.22	32.35±20.70	47.19±32.51	15.58	0.000
PLT(/L)	226±46.25	207±56.40	186.90±100.35	5.73	0.004

Table S2

Stratum analysis of different parameters in all subjects elder than 50-year-old

Indices	healthy(49)	HBV(24)	HCC(110)	F	P
GA	13.55±1.26	12.79 ±1.79	16.12±4.36	15.51	0.000
TC (mmol/L)	4.76±1.12	4.70±1.13	4.01±0.90	11.72	0.000
TG (mmol/L)	2.80±1.57	1．81±1.48	1.03±0.52	15.54	0.005
LDL (mmol/L)	2.83±0.54	2.70±0.87	2.46±0.74	43.65	0.000
HDL (mmol/L)	1.42±0.36	1.35±0.39	1.34±0.31	2.45	0.361
MDA (nmol/mL	9.60±5.47	9.96±3.12	12.20±9.13	0.114	0.105
TAOC (U/ml)	18.35±14.10	5.76±2.73	14.08±5.49	17.32	0.000
TBIL(μmol/L)	13.23±5.54	16.46±9.71	17.78±17.30	1.749	0.177
TP(g/L)	77.26±3.02	79.54±5.18	71.39±6.35	33.07	0.000
ALB(g/L)	46.76±2.30	47.8±2.59	40.92±4.22	65.50	0.000
ALT (U/L)	21.14±8.78	30.75±16.40	58.42±48.56	6.90	0.001
PLT(/L)	226.95±54.79	193.00±59.62	148.00±70.00	22.90	0.000

Table S3

Strata analysis of HDL, MDA,TBIL in HCC patients at different ages

Indices	<50 (X±SD)	⩾50 (X±SD)	t	P
HDL (mmol/L)	1.31±0.34	1.35±0.33	1.40	0.160
MDA (nmol/mL)	11.52±6.33	11.72±7.71	0.28	0.780
TBIL(μmol/L)	14.07±6.65	15.96±13.54	1.810	0.070

Conclusion 1: After stratified by age, the results were similar to what we have got from the whole subjects.
