# Supplementary material for: Association between metabolic abnormalities and HBV related hepatocelluar carcinoma in Chinese: A cross-sectional study
Source: Nutr J. 2011 May 15;10:49. doi: 10.1186/1475-2891-10-49 (PMC3118330; doi:10.1186/1475-2891-10-49)
Supplement: Additional file 2 — Stratum analysis of parameters in accordance with gender. After stratified by gender, the results were similar to what we have got from the whole subjects. [file 1475-2891-10-49-S2.PDF]

Additional file 2(Table S4- Table S5)

Table S4

Strata analysis of different parameters in HBV-related HCC subjects between male and female

Indices	Male(155)	Female(24)	t	p
GA	15.61±0.31	15.33±3.63	0.33	0.74
TC (mmol/L)	0.89±0.07	4.24±0.87	-1.673	0.096
TG (mmol/L)	0.99±0.46	0.88±0.37	1.109	0.269
LDL (mmol/L)	2.43±0.72	2.58±0.82	-0.898	0.371
MDA (nmol/mL	12.78±8.54	11.51±5.53	0.703	0.483
TAOC (U/ml)	13.98±5.28	14.52±5.27	-0.465	0.642
TBIL(μmol/L)	15.88±10.86	19.77±17.73	-0.665	0.513
TP(g/L)	71.13±5.92	71.86±6.88	-0.54	0.59
ALB(g/L)	40.96±4.00	40.55±3.76	0.465	0.642
ALT (U/L)	56.05±39.33	41.22±18.17	1.019	0.31

Table S5

Stratum analysis of different parameters in all subjects of male

Indices	healthy (118)	HBV(66)	HCC(155)	F	P
GA	13.53±1.34	11.70±1.85	15.61±3.84	53.89	0
TC (mmol/L)	4.62±0.94	4.74±1.02	3.91±0.89	37.38	0
TG (mmol/L)	2.28±3.38	1.68±1.06	0.99±0.46	20.61	0
LDL (mmol/L)	2.93±0.69	2.87±0.82	2.43±0.72	24.50	0
MDA (nmol/mL	9.1±5.42	9.83±3.59	12.78±8.54	14.43	0
TAOC (U/ml)	19.68±7.27	6.24±3.22	13.98±5.28	38.874	0
TBIL(μmol/L)	13.39±5.58	16.46±9.86	15.88±10.86	3.572	0.03
TP(g/L)	77.63±3.21	78.25±4.47	71.13±5.92	76.097	0
ALB(g/L)	47.33±2.29	48.43±2.25	40.96±4.00	199.82	0
ALT (U/L)	26.20±15.87	34.97±18.86	56.05±39.33	14.00	0

Conclusion 2: After stratified by gender, the results were also similar to what we have got from the whole subjects.
