# Supplementary material for: Association between metabolic abnormalities and HBV related hepatocelluar carcinoma in Chinese: A cross-sectional study
Source: Nutr J. 2011 May 15;10:49. doi: 10.1186/1475-2891-10-49 (PMC3118330; doi:10.1186/1475-2891-10-49)
Supplement: Additional file 3 — The associaton between tumor size and TG or GGT. Correlation analysis between tumor size and TG. The tumor size was negatively associated with TG and positively related to GGT. [file 1475-2891-10-49-S3.PDF]

Additional file 3(Figure S1)

Figure S1. The associaton between tumor size and TG or GGT. Correlation analysis between tumor size and TG. The tumor size was negatively associated with TG and positively related to GGT.

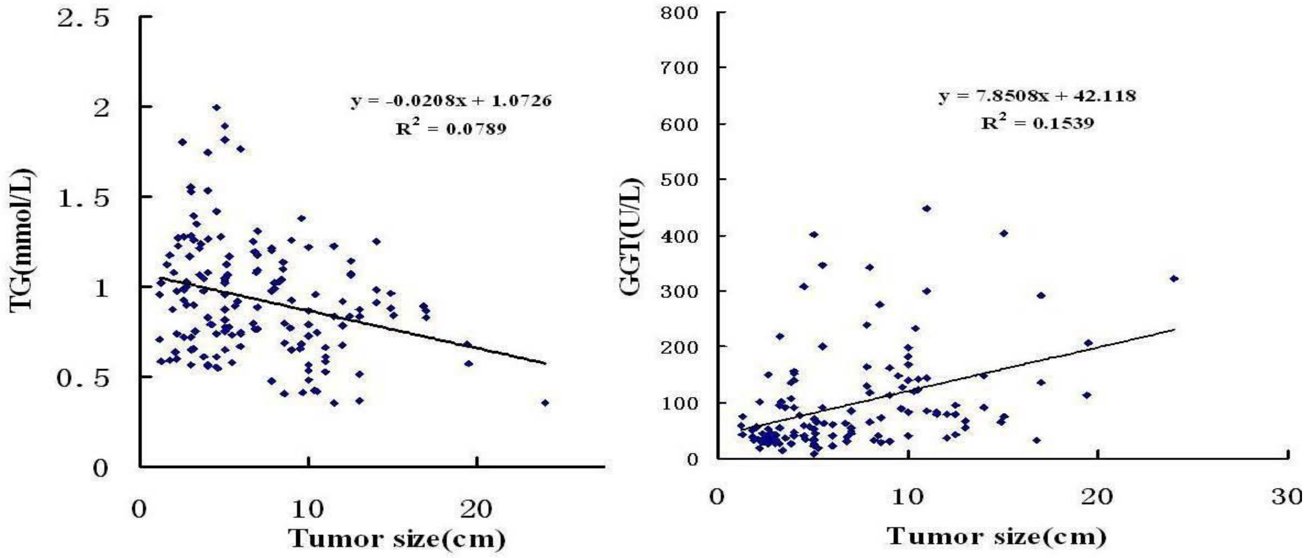

Figure S1
